# Unmet Health Care Needs of the Older Population in European Countries Based on Indicators Available in the Eurostat Database

**DOI:** 10.3390/healthcare11192692

**Published:** 2023-10-08

**Authors:** Ewa Kocot

**Affiliations:** Health Economics and Social Security Department, Institute of Public Health, Faculty of Health Sciences, Jagiellonian University Medical College, Skawinska 8, 31-066 Krakow, Poland; ewa.kocot@uj.edu.pl; Tel.: +48-12-4332-818

**Keywords:** unmet health care needs, older population, access to health care, age groups, waiting lists

## Abstract

Access to healthcare may affect the health of the population, especially older people. The aim of this study is to analyze the reasons and factors influencing the unmet healthcare needs (UHCN) of the older population in the context of differences between age groups for 28 European countries. A self-reported UHCN indicator obtained from Eurostat database was used. The share of people with healthcare needs reporting distance/transportation issues was significantly different in the younger and older groups, as well as in age groups within the older population. The differences in other reasons were not so considerable. Problems with UHCN were observed more often in the older population with lower rather than with higher income and with more severe activity limitations rather than with none/moderate limitations (differences statistically significant, except for income for 75+). In most countries, the UHCN dependence on income/activity limitation is higher in the age group of 15–64 than for the older population. To plan/introduce/monitor appropriate, tailored actions for improving healthcare access for the older population, a detailed analysis of the UHCN prevalence, reasons, and determinants in this age group is needed; it is insufficient to analyze only the population as a whole. Additionally, the group of older people is not homogeneous in terms of UHCN.

## 1. Introduction

Adequate access to health care is considered one of the determinants of population health [1,2]. Although it is estimated that medical care (access and quality) accounts for only 10–20% of modifiable health determinants, actions introduced to improve access to health care influence the health of individuals and the population [3,4,5].

Due to the progressive aging of the population (the share and number of people aged 65+ is still growing [6]), maintaining this segment of the population in the best possible health condition is becoming more and more important, as they play an increasingly important role in societies. Because life expectancy in the older age is shorter, health determinants, the influence of which becomes apparent in the long run, may be less important for the health of older people. At the same time, health usually deteriorates with age, and older people often suffer from a multi-morbidity, so their health care needs are higher. As a consequence, access to proper health care becomes relatively more significant for older than for younger people. Making optimal allocation decisions is not possible without basing them on reliable evidence [7]. Understanding the factors which affect access to health care for this group is especially important during these decisions [8].

Many different indicators may be used to evaluate access to health care. Some examples include measures of health care utilization (e.g., the annual number of medical consultations per inhabitant) or indicators related to health care resources: workforce (e.g., the number of physicians/nurses per 100,000 inhabitants), facilities (e.g., the number of inhabitants per one hospital bed) or technologies (e.g., magnetic resonance imaging units per 100,000 inhabitants) [6]. However, measures of utilization or resources do not capture issues faced by people who do not use a given health care service despite the need to do so; thus, they can be used only as proxy indicators of how well these needs are met [9]. This kind of information—about health care which was not received appropriately—is provided by indicators of unmet health care needs (UHCN) [10,11,12]. This type of indicators, usually based on self-reported data, is used most often as a proxy for assessing access to health care services [13].

In European Union (EU) countries, a core set of health services is provided to nearly all residents, but even if widespread health care access is formally ensured, UHCN still exist in every country [14]. There are many reasons for this issue, the main ones being financial problems, long waiting times, and the distance to health facilities (transportation problems) [15]. However, studies regarding UHCN have been conducted more often in the United States and Canada than in Europe, and a relatively small amount of research relates to a large group of European countries or wider international settings [16,17,18,19,20]. When analyzing the determinants of UHCN in Europe, some authors have taken the age factor into account [18,20], but studies focused on the older population are much more rarely conducted and are usually placed in a specific context (e.g., in relation to the COVID pandemic [21,22,23,24]; nursing care [25]; primary care [26]; depression [27]). As a result, there is a knowledge gap regarding general UHCN differences between older and younger people, considering reasons behind UHCN and associated factors. Additionally, to the author’s best knowledge, the heterogeneity of the older population is rarely included in research. To diagnose the situation and provide directions to improve current and future access to health care, it is worth paying special attention to health care access issues in relation to older people.

Due to this rationale, the general aim of this study is to analyze the reasons and factors influencing the UHCN of the older population in the context of differences between age groups—(a) between younger and older populations (15–64 vs. 65+) and (b) within the older group (65–74 vs. 75+)—based on the example of 28 European countries. The specific research questions are: (1) what reasons and factors influencing UHCN are more/less important for older than for younger populations? (2) Is the group of older people homogeneous in terms of the reasons and factors influencing UHCN? The year of focus for our analysis is 2019; the UHCN indicator obtained from the European Health Interview Survey and published in the Eurostat database was used. The study was conducted for 26 EU countries + Iceland and Norway (this choice was based on data availability). 

## 2. Materials and Methods

### 2.1. Framework of the Study

**Figure 1:**
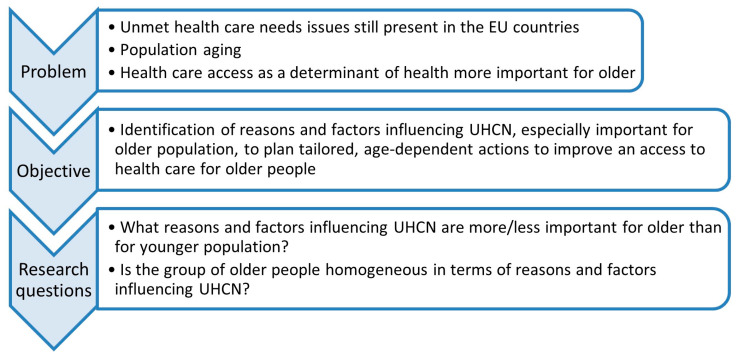
The general concept of the study.

The overall concept of the study is presented in Figure 1.

Many authors refer to the definition of UHCN formulated in 1976 by Carr and Wolfe [11,16,19,20,28,29,30]. They generally define UHCN as “the absence of any, or of sufficient, or of appropriate care and services” [31] (p. 418). Their operational definition indicates that UHCN are the differences between the services judged necessary to deal with a given health problem and the services actually received [31]. A similar definition of unmet health care needs was presented by Sanmartin et al.: “a difference between healthcare services deemed necessary to deal with a particular health problem and the actual services receive” [30] (p. 16). The above definitions are very general and covers many possible concepts of UHCN. Allin, Grignon, and Le Grand (2010) distinguished five categories of UHCN, highlighting the complex and multi-dimensional character of this concept: (1) unperceived unmet needs: an individual does not know about his/her need, although it is present; (2) subjective, chosen unmet needs: an individual is aware of needs, but due to various reasons chooses not to demand the health services, despite the fact that they are available; (3) subjective, not-chosen unmet needs: an individual is aware of needs and they are not met due to reasons beyond his/her control; (4) subjective, clinician-validated unmet needs: an individual is aware of needs and receives health care services, but they are clinically judged to be inappropriate; (5) subjective, unmet expectations: an individual is aware of needs and receives health care services, but they are judged by him/her to be inappropriate [11]. 

Adapting Andersen’s influential behavioral model regarding access to health care, factors affecting unmet needs can be divided into three groups: (1) predisposing factors: socio-economic and demographic factors, like age, sex, education level, marital status, and immigration status; (2) enabling resources: individual-level factors (e.g., having a regular doctor, household income, place of residence) and community-level factors (e.g., density of family and specialist physicians); and (3) health needs factors: health status and behaviors [12,32]. This model is the most commonly used in studies related to UHCN in the older population [8,33,34,35]. Models used in studies are also frequently various modifications of the Levesque conceptual framework of access dimensions, based on a constructed path: health care needs—health care seeking—health care reaching—health care use—health consequences [8,36,37]. 

According to Allan and Ammi (2021), while some UHCN may be due to systemic reasons (e.g., overlong waiting lists, high costs of care), others are caused by personal reasons (e.g., individual preferences, constraints not directly related to the health care system) [12]. Another typology of reasons was presented by Chen and Hou [38] and used in widely in research, e.g., [10,38,39]. They are classified into three categories: (1) availability: services are not available when required (e.g., overlong waiting lists, lack of facilities in area); (2) accessibility (e.g., costs of services, transportation issues); (3) acceptability: services are available, but not used due to personal preferences (e.g., fear of the doctor, busyness). Results regarding the main reasons of UHCN are not conclusive in the literature and depend on the method of analysis and the country (or even a country region), and may also change in time and with age [10,12,15,40].

**Figure 2:**
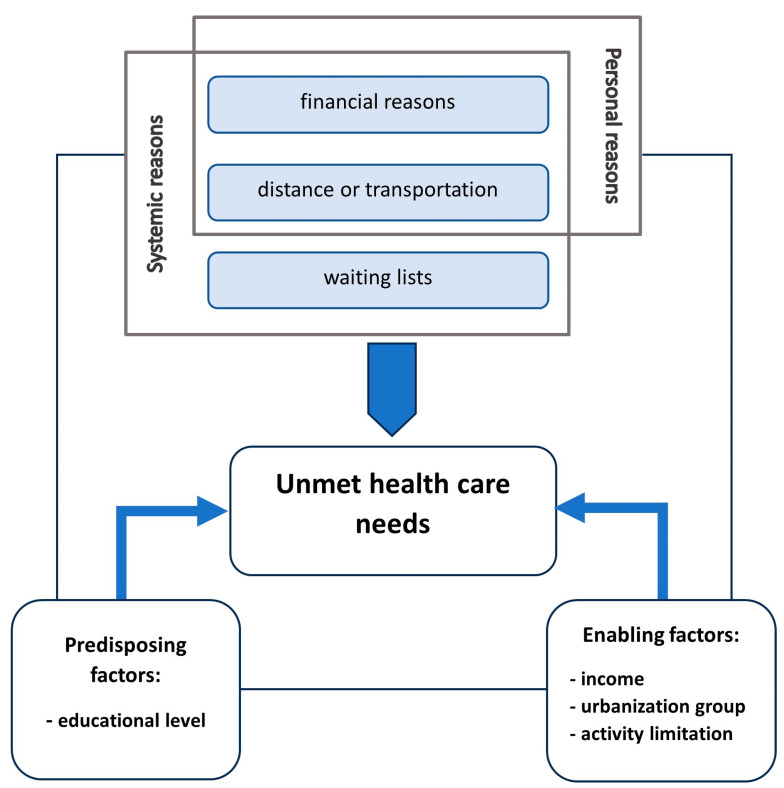
The framework of the research.

In the presented analysis, based on the Andersen’s model and the division of UHCN causes used in literature (mentioned above), taking into account the limitation related to the data availability, the following framework of research was adopted (Figure 2): 

Three reasons behind UHCN are included in the analysis: financial reasons, distance or transportation, and waiting lists. While overlong waiting lists belongs to the group of systemic reasons of UHCN, financial problems may be classified as systemic reasons (e.g., if they are related to high costs of care) or personal reasons (e.g., problems related to a low family income). Distance or transportation issues can also be systemic or more personal (problems caused by a low density of health care facilities and by different types of mobility limitations, respectively). One predisposing factor (educational level) and three enabling factors (income, urbanization, and activity limitation) were included in the study. The third group of factors (health need factors) is out of the scope of this analysis.

Looking at the categories of UHCN defined by Alli, Grignon, and Le Grand (described above), the presented analysis concerns mainly the second and third UHCN type: subjective, chosen, and not-chosen unmet needs. 

### 2.2. Materials

Countries included in the analysis were selected based on data availability (26 EU countries (without Belgium) + Iceland and Norway). This is a cross-sectional study; the year of the analysis is 2019. The year 2019 is the last year of data availability, but also the last possible year to analyze the meeting of health care needs not affected by specific factors related to the pandemic.

The share of older people (aged 65 and above) in the analyzed countries ranges from 14.8% in Luxembourg to 23.8% in Italy (2022) but will continue to grow (range from 20.5% (Iceland) to 35.5% (Greece) in 2050), with the highest growth in Greece: 12.8 percentage points (pp)) [6]. Health care systems in all analyzed countries are financed mainly from public sources (government and social health insurance). However, the share of these financing schemes varies substantially between countries: from 55.5% in Cyprus to 85.7% in Norway. The share of out-of-pocket payment ranges from 9.5% in France to 37.8% in Bulgaria (2019 data) [6].

The analyses were prepared based on the UHCN indicator, obtained from the Eurostat database [6]. Eurostat is the statistical office of the European Union, created to prepare and publish statistics and indicators on the EU countries. The data provided by Eurostat allow for extensive analyses, including comparisons between countries and regions. 

There are two indicators regarding UHCN available in the Eurostat database. They are calculated based on self-reported UHCN information obtained from two questionnaire surveys: (1) the European Health Interview Survey (EHIS indicator) and (2) the EU Statistics on Income and Living Conditions (EU-SILC indicator). Both indicators are entirely subjective, but they vary in many points (a comparison of the indicators is presented in Table S1). To achieve the aim of this study, the EHIS indicator was used for analyses, as it is more generally defined and takes into account the level of health needs (detailed methodological information about EHIS can be found here: [41]). As the values of the EU-SILC indicator are presented in relation to the whole population (not the population with health care needs), these values are very low. Rounding them to the first decimal place (as is done in the Eurostat data) results in missing small differences, if any exist. The questions from the European health Interview Survey related to indicators used in the analysis are presented in Questionnaire S1 (Supplementary Materials). 

The target population in the EHIS included individuals living in private households only, excluding persons living in institutions and collective households. Depending on country, the sampling unit was the dwelling, the household, or the individual; multi-stage stratified, systematic (cluster), or single stage sampling was used. The achieved sample size was 321,696 in total (the sum of all countries samples; detailed information for countries is presented in Table S2), while the total population aged 15 and more in the analyzed countries was equal to 373,829,948 in 2019 [6]. The ratio of the achieved sample size to minimum effective sample size ranged from 0.39 to 2.79 (depending on country, lower than one in five countries). It was confirmed by the Eurostat experts that the results of the quality assessment meet the expectations regarding to the quality of the survey, implementation, and performance, and an overall good comparability level across countries was achieved [41].

The analyses were conducted by age (using age groups 15–64, 65+, 65–74, and 75+), reasons for UHCN (financial reasons, distance or transportation, waiting list), and population characteristics (income, educational level, urbanization group, and activity limitation level). The age groups were selected as above to allow the analysis of differences between younger (15–64) and older (65+) population, as well as analysis within the group of older people (65–74 and 75+). This division of the older population group was determined by data availability. 

The data used in the study was only secondary, aggregated data, without any personal information included. All the data is publicly available in the online Eurostat database [6].

### 2.3. Methods

Descriptive analyses, as well as statistical tests, were conducted in the course of the research. The main indicator of UHCN used in the analysis (EHIS indicator) was based on self-reported data and calculated for a given country and age group as follows:

$${UHCN}_{c,a}=\frac{P_{UHCN,c,a}}{P_{N,c,a}}$$

where *P_UHCN_*_,*c*,*a*_—number of population aged 15+ in a country *c* and age group *a* reporting unmet health care needs in the previous 12 months prior to study; *P_N_*_,*c*,*a*_—number of population aged 15+ in a country *c* and age group *a* reporting health care needs in the previous 12 months prior to the survey. 

The indicator value is presented as percentages and may be interpreted as a share of population in need of health care whose needs have not been met.

Apart from the basic indicator obtained from the Eurostat database, two additional indicators were calculated:

(1) The share of people reporting a given reason of UHCN, calculated for each age group and country as follows:
  $$share\left(r\right)=\frac{{UHCN(r)}_{c,a}}{{UHCN}_{c,a}}$$
  where *r*—UHCN reason; *c*—country; *a*—age group.

Data used for the indicator calculation are included in Table S3.

(2) The range of UHCN indicator values for income groups/educational levels/urbanization groups/activity limitation levels, for each age group and country, calculated as follows (exemplary for income groups):
  $${range\;I}_{c,a}={\max I}_{c,a}-{\min I}_{c,a}$$
  where *I_c_*_,*a*_ is a set of five quintile income groups for a country *c* and age group *a*.

The analyses focused on differences in reasons and factors influencing UHCN between age groups: (1) 15–65 and 65+, and (2) 65–74 and 75+. 

Depending on whether the assumptions were met, parametric (one-way ANOVA, *t*-test) or non-parametric (Mann–Whitney U test) statistical tests and measures were used. The variables were checked for normal distribution and homogeneity of variance using the Shapiro–Wilk test and Levene’s test, respectively. Analyses were conducted using the IBM SPSS Statistics 28. The significance level was set at *p* < 0.05.

## 3. Results

### 3.1. UHCN by Countries and Age Groups

The total level of UHCN in the analyzed countries is presented in Table 1 for a general overview.

In more than half of the countries (16 out of 28), the proportion of the population with UHCN was higher in the group from 15–64 than in the group 65+ and this difference exceeded 10 pp in six countries (Germany, France, Norway, Iceland, Luxembourg, and Denmark). The absolute difference of UHCN values between the older (65+) and the younger population (15–64) ranged from 0 to 15.9 percentage points (pp), depending on the country. In three countries (Poland, Croatia, and Romania), the value was much higher (over 10 pp) in the older population (65+) than in the younger population (15–64). The smallest differences between the considered age groups were in Lithuania (no differences) and Latvia (0.4 pp). In most countries, there were no major differences in the UHCN indicator value inside the older population group (between age groups 65–74 and 75+)—over 85% of the countries had an absolute difference of less than four pp (Figure 1, Table S2).

### 3.2. UHCN by Age and Reason

In almost every country (except three), more than a half of the people with UHCN in every age group had a problem with the time in which services were provided. The share of the UHCN related to waiting lists differed depending on the country, ranging from 24% to 96% for the age group from 15–64 and for the age group 65+ from 26.7% to 91.3%. In over 60% of countries (16 out of 26), a higher share of UHCN was caused by waiting lists in the 15–64 age group than in 65+, but statistical significancy was not confirmed for differences between these age groups (Table 2). 

Financial reasons for UHCN were reported more frequently in the younger group (15–64) than in the older one (65+) in the vast majority of countries (82%), and in some of them, the difference between these age groups was quite high, even exceeding 30 pp in Finland. The mean for the younger group was 58.9, while for the group aged 65 and more, it was 49.3. This difference was statistically significant (t-statistic = 2.18; *p* = 0.034) (Table 2). 

Distance and transportation issues were, in general, reported less frequently as a reason for the UHCN than the financial and waiting list reasons. In two cases only (France and Iceland), this reason was mentioned more often in the younger group (15–64) than in the older one (65+). As in the case of financial reasons, this difference was statistically significant (Mann–Whitney U test: Z = −3.19; *p* = 0.001) (Table 2).

The statistical tests did not confirm a significant difference in the share of the UHCN caused by financial issues and by waiting lists between the age groups 65–74 and 75+ (Table 2). In the case of distance or transportation as a reason for UHCN, there were considerable differences between the 65–74 and 75+ age groups: in the former, this cause was indicated, depending on the country, in 1.3% to 33.8% of cases, while in the latter, it was indicated in 8.9% to 50.2% (with medians of 13% and 22%, respectively). In the vast majority of countries (24 out of 28, 86%), people aged 75+ indicated this reason much more frequently than people in the age group 65–74, and the difference even reached 27 pp (Luxembourg). The difference between these age groups was statistically significant. However, in both older age groups (65–74 and 75+), the distance or transportation reason was reported much less frequently than the financial and waiting list causes (Table 3).

### 3.3. UHCN by Predisposing Factor

In the case of educational levels, the differences in the value of the UHCN indicator within individual countries were smaller than in the case of income groups.

Looking at the mean values, a decrease in UHCN could be observed with an increase in educational level. However, the differences were small, and the statistical analysis did not confirm a significance of differences between groups of various educational levels (Table 4 and Table S4).

**Figure 3:**
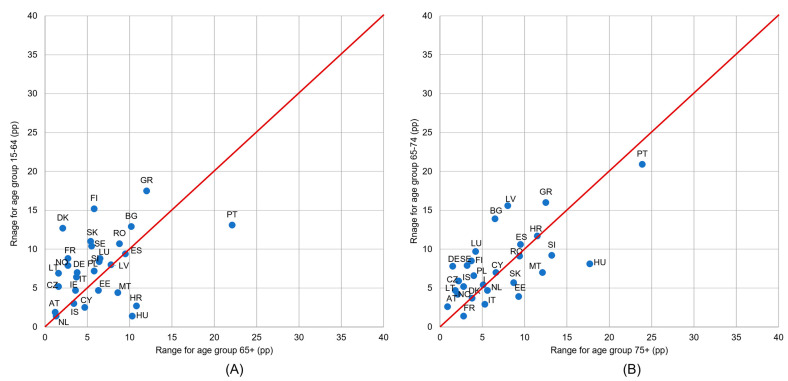
Ranges of unmet health care needs (UHCN) indicator values in the set of three educational levels: (**A**) for age groups 65+ and 15–64 and (**B**) for age groups 75+ and 65–74. Notes: range is a difference between maximum and minimum in the set of values for three educational levels, calculated for each country and age group; if a country is marked on the red line, it means that differences in UHNC between educational levels are equal for both age groups; if a country is above the red line, it means that differences between educational levels are higher for the age group on the vertical axis; a large country distance from the red line means that the differentiation according to education varies considerably between age groups.

It can be seen that the presented countries were more concentrated along the red line than in the case of income groups analysis (Figure 3). This means that differences in UHCN between educational level groups were rarely dependent on age (for two analyzed pairs of age groups).

### 3.4. UHCN by Enabling Factors

In this section, UHCN are analyzed according to three characteristics: income group, degree of urbanization, and level of activity limitations.

#### 3.4.1. Income Groups

In the vast majority of countries and age groups, lower income was associated with a higher value of the UHCN indicator. This trend was evident when looking at the means for the analyzed group of countries for all groups of population (Table 5 and Table S5).

For the age groups 15–64, 65+ and 65–74, differences in the value of the UHCN indicator between income groups were statistically significant. Post hoc analysis indicated this significance mainly for the first quintile group, compared to other groups (except for the second quintile group for the age 15–64 and 65+). For the age group 75 and over, there was no justification for rejecting the null hypothesis of no difference between the income groups (Table 5).

**Figure 4:**
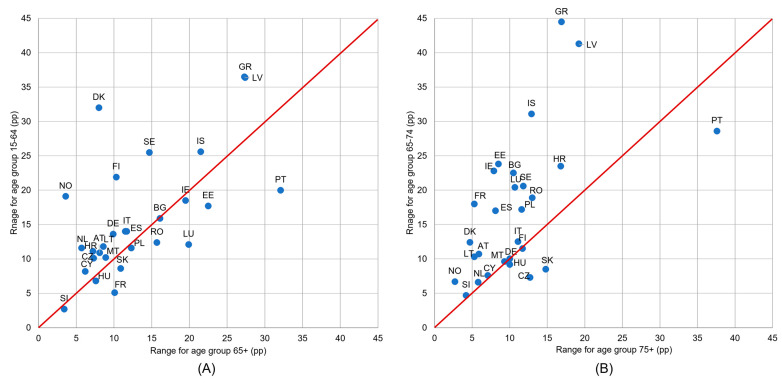
Ranges of unmet health care needs (UHCN) indicator values in the set of five income groups: (**A**) for age groups 65+ and 15–64 and (**B**) for age groups 75+ and 65–74. Notes: range is a difference between maximum and minimum in the set of values for five quintile income groups, calculated for each country and age group separately; if a country is marked on the red line, it means that differences in UHNC between income groups are equal for both age groups; if a country is above the red line, it means that differences between income groups are higher for the age group on the vertical axis; a large country distance from the red line means that the differentiation according to income varies considerably between age groups.

The difference magnitude between income groups depended on the country and on the age group. Figure 4 shows a comparison of the UHCN values ranges (a difference between the highest and the lowest value in the set of income groups) between age groups 65+ and 15–64 (graph A); 75+ and 65–74 (graph B). In many more countries, higher differentiation according to income could be observed in the age group 65–74 than in the age group 75+ (only four countries are located under the red line). Similarly, in most countries, higher differences in income are found in the younger age group, 15–64, than in the older one, 65+ (Figure 4).

#### 3.4.2. Urbanization Groups

The differences of the UHCN between people from different urbanization groups were small and statistical significancy of differences was not confirmed for any age group (Table 6). Looking at the mean values, a slightly higher level of the UHCN in cities and lower in rural areas could be observed. (Table 6 and Table S6).

**Figure 5:**
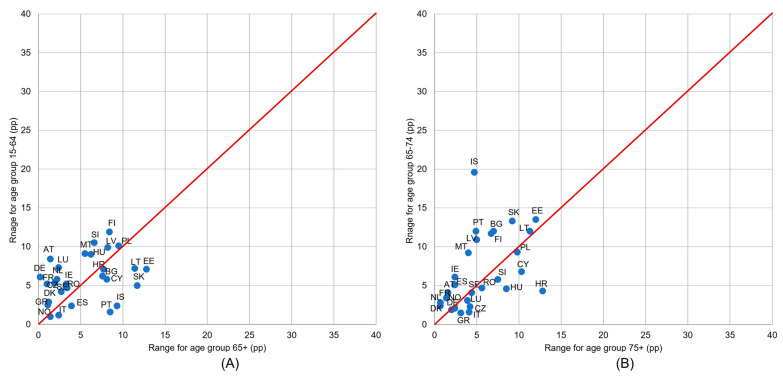
Ranges of unmet health care needs (UHCN) indicator values in the set of three urbanization groups: (**A**) for age groups 65+ and 15–64 and (**B**) for age groups 75+ and 65–74. Notes: Range is a difference between maximum and minimum in the set of values for three urbanization groups, calculated for each country and age group separately; if a country is marked on the red line, it means that differences in UHNC between urbanization groups are equal for both age groups; if a country is above the red line, it means that differences between urbanization groups are higher for the age group on the vertical axis; a large country distance from the red line means that the differentiation according to urbanization varies considerably between age groups.

In more than half of the countries, the diversity of the UHCN depending on the level of urbanization was bigger in younger age groups: in the group 15–64 bigger than in 65+, and in the group 65–74 bigger than in the group 75+ (Figure 5). Comparing Figure 5 with Figure 4 it can be seen that in the case of variations by urbanization groups there were far less countries with as large differences between age groups as is seen in the case of the income level (for many more countries the distance from the red line is considerably larger in Figure 4).

#### 3.4.3. Activity Limitations

There were substantial differences in the intensity of UHCN issues between groups with various activity limitation levels. It is clearly visible in Table 7 that a level of activity limitation was positively associated with a level of UHCN, regardless of age group: the mean for the group with severe limitations was at least twice as high as the value in the group without any limitations. For all analyzed age groups, these differences were statistically significant; post hoc analysis indicated this significance for each pair of activity limitation levels (Table 7 and Table S7).

**Figure 6:**
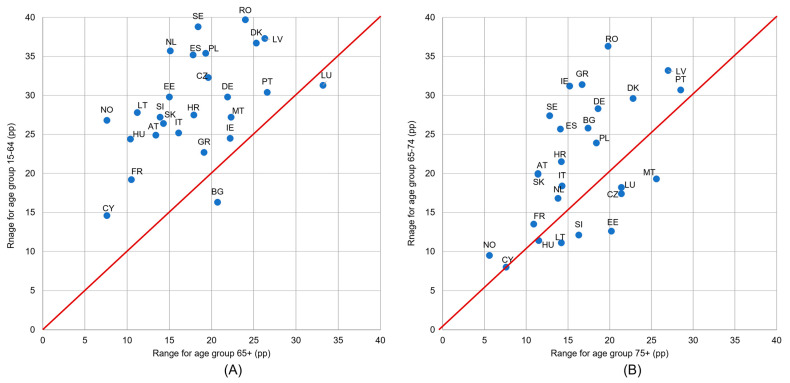
Ranges of unmet health care needs (UHCN) indicator values in the set of three activity limitation levels: (**A**) for age groups 65+ and 15–64 and (**B**) for age groups 75+ and 65–74. Notes: range is a difference between maximum and minimum in the set of values for three activity limitation level, calculated for each country and age group separately; if a country is marked on the red line, it means that differences in UHNC between activity limitation levels are equal for both age groups; if a country is above the red line, it means that differences between activity limitation levels are higher for the age group on the vertical axis; a large country distance from the red line means that the differentiation according to activity limitation varies considerably between age groups.

The data in Figure 6 indicates clearly that differentiation according to activity limitation level was much stronger in the age group from 15–64 than in the 65+ group, and in the age group from 65–74 than in the 75+ group. There were considerable differences between age groups in many countries, especially for the first compared pair of age groups (15–64 and 65+).

## 4. Discussion

The general aim of this study was to analyze the reasons and factors influencing the UHCN of the older population in the context of differences between age groups, based on the example of 28 European countries. This study provides a first general look at the issue of differences between older and younger groups of people in UHCN prevalence by reasons and potential relationship with various factors, as well as within the older population. The novelty of the study compared to existing studies is the analysis of differences between younger and older populations in the context of reasons and factors influencing UHCN, while other studies usually analyze the older group separately or compare with the younger group only in terms of a total UHCN level. This study also provides information about differences within the older population, while existing research usually treat this group as homogeneous. Additionally, the analysis includes 28 countries, while most studies focus on one country.

According to the framework presented in Figure 2, the results are discussed below in three groups: differences related to (1) reasons; (2) predisposing factors; (3) enabling factors.

### 4.1. Reasons of UHCN

Looking at the data, it is clear that, in most countries, the UHCN of the older population were caused by overly long waiting time less often than for the younger population. Although these differences were usually not substantial and their statistical significance was not confirmed in the analyzed countries, this difference can matter on the level of an individual country. The differences in the prevalence of waiting time issues between age groups (younger and older, and within the older group as well) may be related to differences in the type of health services needed in these groups. A deeper analysis would be needed to recognize this in detail.

A significantly lower share of the UHCN for the population aged 65+ than for those aged from 15–64 was due to financial problems. A possible explanation could be the existence of various program and facilitations of health care financing aimed at supporting older people that have been introduced in some countries (e.g., an extensive list of free medicines, mainly for chronic and cardiovascular diseases, for older patients in Poland [42]).

It is not surprising that the share of people with UHCN who indicate a distance and transportation reason was higher in the older population (65+) than in the younger population (15–64) (statistical significancy confirmed), as older people need help with transport in order to reach a health care facility much more often (at least one-third of older people reported unmet needs related to travel in general) [43]. An even bigger difference depending on age could be observed for the age group 65+: people aged 75+ usually reported distance and transportation problems much more frequently than the group aged 65–74, and this difference was also statistically significant. Distance and transportation issues, presented in the EHIS survey as one group of reasons, can in fact be seen as a health care system reason, but also as a personal reason for UHCN. Health care facilities can be, for example, concentrated in urban areas, and the distance that people living in rural areas must travel to see a doctor may be a significant barrier, even for young and active people. This may be especially true in the case of highly specialized health care. However, for the older population, the results showed only slight differences in UHCN depending on urbanization group; furthermore, people living in rural areas reported, on average, a lower level of UHCN than those living in cities and towns. For older people, UHCN caused by distance and transport issues are probably more related to mobility limitations than distance. This may be confirmed by the fact that in all analyzed groups of older people, there were meaningful differences in UHCN prevalence depending on activity limitation level, and the UHCN indicator value was higher when greater limitations in activity were reported. The shown differences in reasons of UHCN between age groups are consistent with the results of the research of Allan and Ammi (2021), showing that the reasons for UHCN change with age [12].

### 4.2. UHCN Prevalence Association with Predisposing Factors

The study did not show major differences in UHCN depending on educational level and did not confirm a statistical significance of them, regardless of the analyzed age group. However, the results seemed to indicate a relatively larger association between educational level and UHCN in the 15–64 group than in the group aged 65+ (and similarly in 65–74 than in 75+). It is possible that bigger differences would be seen when looking at UHCN of the first type as defined by Allin, Grignon and Le Grand (2010): unperceived unmet needs [11]. The frequency of this category’s occurrence may depend on factors that may potentially vary between age groups: one’s awareness of what health is and what it is not, on health literacy (thus indirectly on education level). However, self-perceived UHCN indicators do not refer to this type of UHCN. After analyzing the results of some European studies, Ramos et al. (2019) stated that there are mixed results concerning the association between education level and UHCN [17]. In Canadian research, it was found that people with higher education levels more often reported UHCN [12,44]. On the contrary, in the research of Fjaer et al. (2017) and Chen and Hou (2002), no association was found [20,38]. The study on European countries of Jürges and Stella indicated that low-educated individuals were more likely to suffer from UHCN, while highly educated people reported UHCN less often [45]; similar results were presented for Turkey [46]. However, in all mentioned studies, the potential result differences related to age are not analyzed.

### 4.3. UHCN Prevalence Association with Enabling Factors

Substantial UHCN inequalities between income groups were more often observed in the age group from 15–64 than in those aged 65+, and in the age group from 65–74 than in those aged 75+ (Figure 4). In 61% of the analyzed countries, a financial reason for UHCN was reported less frequently among people aged 75+ than among those from 65–74; this could suggest that, in fact, the financial possibilities of potential patients are of less importance at the oldest ages. The statistical analysis also confirmed that UHCN differences by income groups were significant in groups from 15–64, 65+, and from 65–74, but not in the age group 75+.

Reeves, Mckee, and Mackenbach (2017) indicated in their research that, among older people, greater public pension entitlement (resulting in higher income level) is correlated with reduced UHCN, but this association is seen only in countries with a relatively high out-of-pocket expenditure level [47]. This can confirm the suggestion of Fjaer et al. (2017), regarding the association between income and UHCN, that people with higher income can more easily bypass waiting lists [20]. A second potential explanation indicated in this study is that low-income groups are more affected by a fear of income loss and are less able to take time off work. On the contrary, Fiorillo (2020) found in his study that people with high income had a higher probability of UHCN due to time constraints [48]. This finding would partly justify the lower sensitivity of UHCN to income in the oldest age group (75+), as people of this age usually do not work and their time constraints are smaller. On the other hand, Jürges and Stella (2019) found in their study that, in general, a lower probability of persistent UHCN is observed among employed people (also Westin et al.) [45,49], but this result was based on the analysis of the total population and is hard to interpret in the context of older age groups.

In most analyzed countries, there were only small differences in UHCN depending on urbanization group, and this was the case in all analyzed age groups (no statistical significance). This is in line with Chen and Hou’s study (2002) confirming that a relationship between the type of residence area (rural or urban) and UHCN was not significant [38]. A slightly higher level of UHCN could be observed in cities than in rural areas, and these differences were, in most countries, slightly more noticeable in the age group from 65–74 than in those aged 75+.

In the vast majority of countries and age groups, the analysis showed an increasing prevalence of UHCN with increasing activity limitation levels, and the differences were significant for all older age groups. The differences according to the activity limitation were clearly higher in younger age groups compared to older ones (respectively: in 15–65 than in 65+; in 65–74 than in 75+) (Figure 6). Other studies confirmed similar results, for example related to disability in European countries [45]; however, this result was obtained without checking for differences in age groups. There was also a negative relationship confirmed between UHCN prevalence and health, but this was also not verified by age groups [18,48].

While the UHCN indicators are usually based on self-reported data, cultural factors, individual expectations, and personal experiences may also influence the outcome. If people anticipate being well treated and are in the proper environment, they are more willing to seek help [17]. The results of the study by Röttger et al. (2016) indicated that negative experiences (perceived discrimination, unfair treatment) with the health care system were a strong predictor of the UHCN of chronically ill people (this is usually the case in the older population) [50]. The health needs of older and younger people differ, and in many cases, health systems are not designed well for the older population [51]. Elements which are more important for the older group than for the younger population include the biopsychological aspects of health, which should be taken into account during health service provision. However, health workers are often not sufficiently prepared to deal with older people [52]. These issues may be sources of bad health care experiences for older people, resulting in lower health care utilization than needed and unmet health care needs.

### 4.4. Study Limitations

The presented analysis is not free of limitations. The detailed demographic structure of the population was not taken into account, while differences in this structure may potentially affect differences in the UHCN level between age groups. Additionally, health care systems´ financing, and organization rules may create both facilitators and barriers to health care access, different for different age groups—they were not included in this study either. A complex analysis such as this would need much more observation considered to be more reliable (including countries outside Europe), while the current research covers only selected European countries, mostly European Union member states. This may be a cause of a potential bias and limit the possibility of generalization. The analyzed UHCN indicator data, made available by Eurostat, depend on some chosen factors that may relate to inequalities in access to health care (income, education, urbanization and activity limitation). Other factors, including those previously mentioned, may influence the level of UHCN and were not included in this analysis due to data restriction.

The analysis regarding differences inside the older age group was conducted using a division into two age groups: 65–74 and 75+. This division, without separating the “oldest old” group (e.g., 85+), may distort the results, as this group is usually characterized by different health needs patterns: more care, less curative services [53].

Additionally, being based on self-reported, cross-sectional data, this study is subject to some limitations caused by the characteristics of self-reported data and does not allow a deeper causality analysis.

### 4.5. Implications of the Study

The results of the presented study may be useful both for decision makers (in health care, public health, and social sectors) and for researchers.

#### 4.5.1. Implications for Decisions-Makers

As indicated in the overall concept of the study, in order to plan tailored, age-dependent actions to improve health care for older people, the identification of reasons and factors that particularly affect the UHCN level in the older population is needed. Awareness of differences between age groups may indicate areas that need more attention for older than for younger people. For example, looking at reasons for UHCN, the analysis showed that decision makers should pay special attention to the distance and transportation issues of older people, more so than in the case of younger people. The overlong waiting lists and financial problems are relatively more important for the younger population. The results also showed that actions aimed at mitigating educational differences in the older population are unlikely to have a large impact on UHCN inequalities. Looking at UHCN differences related to income group, the analysis showed that there is no indication for policy activities in this context in the group aged 75+; this would be more recommended in the group from 65–74 and especially in the lowest income groups. The differences in activity limitation intensity were significantly associated with the UHCN level regardless of age, but looking at ranges analysis, actions seem to be also more important in the group from 65–74 than those aged 75+. However, the UHCN differences between activity limitation levels were most clearly visible in the younger group, those aged 15–64.

One of the examples of activities affecting access barriers, related especially to the distance and transportation, could be strengthening social support networks. This is an action mainly targeted at older people, who reported the distance and transportation reason for UHCN much more frequently than younger groups. According to the study of Fiorillo (2019), a higher frequency of contact with relatives is associated with a lower probability of UHCN due to distance reasons [48]. A policy decision aimed at improving access to health care may concern, for example, the setting of annual out-of-pocket payment ceilings according to households’ income (such a reform was introduced in Belgium in 2002) [54]. Detailed knowledge regarding the relationship between an income level and UHCN, considering different population characteristics, may be very helpful in making such a decision in a proper way.

#### 4.5.2. Implications for Researchers

In order to make appropriate decisions, policy makers must have access to reliable evidence. The results of this analysis are a clear indication for researchers and entities influencing the dissemination of results that there is a need not only to conduct analyses in age groups but also to make the findings widely available in a disaggregated form.

The study indicated distance and transportation reasons for UHCN as especially important for the older population. However, a deeper analysis should be conducted by researchers, exploring whether this cause is, in the case of the older population, more systemic or personal in nature (systemic reasons for UHCN may be more important for health policy makers’ decisions, while personal reasons may more often indicate specific social/public health activities).

There are studies analyzing the UHCN level depending on age, but without joint analysis considering age and other factors simultaneously. As this study indicates that significant age differences exist in some areas, it would be recommended to conduct deeper future research based on age groups in the following directions: (1) detailed analysis of relationship between a declared reason of UHCN and factors potentially influencing UHCN prevalence; (2) analysis of an interdependence of factors in the context of UHCN prevalence; (3) time-series analysis identifying cause–effect aspects.

The UHCN indicator used in this analysis is based on a self-reported, fully subjective perception of need and delay. The limited accuracy of self-reported data has been shown by many researchers, indicating various forms of potential bias, i.a. response errors [54,55,56,57,58]. The older population group is even more sensitive to problems related to the self-reported study, as they are more often unable to answer questions for health reasons or their answers are less reliable. Future work on the survey methods and content, considering age specificity of respondents, would be advisable. In the context of found differences, it would be useful to consider different types of health care services and needs separately in the survey (e.g., ambulatory/in-patient care, curative care/rehabilitative care/nursing), as well as long-term care.

## 5. Conclusions

Differences in terms of reasons for UHCN and factors influencing them are observed between age groups, as well as within the group of older people. Due to these differences, in order to plan, introduce, and monitor the results of appropriate, tailored actions for improving health care access for the older population, a detailed analysis of the UHCN prevalence, reasons and determinants in this age group is needed; it is insufficient to thus analyze only the population as a whole. Additionally, according to the research results, the group of older people is not homogeneous in terms of UHCN: substantial differences in UHCN within the older population can be observed related to different characteristics like income or activity limitations. Thus, in UHCN analyses, a division of the age group 65+ by age would also be advisable. Even in the cases where a statistical significance of differences in UHCN between age groups were not confirmed, there are individual countries with considerable differences which could be important for decisions in the health policy area.

## Figures and Tables

**Table 1:** The level of UHCN by countries and age groups.

Country	Total UHCN				
	Total	15–64	65+	65–74	75+
Austria	24.9	26.2	20.1	20.8	19.2
Bulgaria	15.9	12.9	22.1	20.7	24.0
Croatia	33.0	28.9	40.3	38.8	41.6
Cyprus	5.8	5.4	7.5	7.8	7.2
Czechia	17.9	16.5	21.9	21.3	22.9
Denmark	33.0	36.8	20.9	20.9	21.0
Estonia	37.1	38.5	32.8	33.7	31.8
Finland	36.3	38.4	30.7	29.4	33.0
France	29.5	32.0	21.5	24.3	17.8
Germany	26.9	29.6	19.4	20.3	18.6
Greece	19.7	18.4	23.5	25.1	21.7
Hungary	22.5	23.2	20.3	21.5	18.7
Iceland	32.5	36.1	21.9	26.8	15.3
Ireland	22.8	23.9	17.8	19.1	15.9
Italy	26.6	25.2	29.9	29.1	30.7
Latvia	36.9	37.0	36.6	41.5	31.4
Lithuania	26.2	26.2	26.2	26.5	25.9
Luxembourg	38.8	41.3	26.6	23.9	33.1
Malta	21.5	20.3	25.7	25.0	26.7
Netherlands	15.4	17.0	10.1	9.9	10.6
Norway	11.7	14.3	2.4	2.9	1.7
Poland	29.6	27.3	37.9	37.8	38.0
Portugal	39.9	39.4	41.4	40.8	42.0
Romania	13.8	10.4	24.7	24.6	24.7
Slovakia	10.9	9.6	15.4	15.1	15.9
Slovenia	28.0	29.7	22.6	23.4	21.5
Spain	19.6	19.9	18.6	19.9	16.9
Sweden	31.7	33.7	26.6	26.6	26.5

Notes: sorted alphabetically by countries.

**Table 2:** The share of people reporting a given reason of unmet health care needs (UHCN): total population, 15–64 and 65+ (%).

Country	Financial			Distance or Transportation			Waiting List		
	Age Group								
	Total	15–64	65+	Total	15–64	65+	Total	15–64	65+
Bulgaria	91.2	88.4	93.7	18.2	16.3	21.3	30.8	34.1	26.7
Romania	87.7	88.5	85.0	12.3	9.6	15.8	26.1	24.0	29.1
Norway	74.4	74.8	50.0	11.1	9.8	25.0	33.3	33.6	37.5
Greece	73.1	76.6	64.3	25.4	17.9	37.0	63.5	59.2	68.5
Finland	70.8	77.3	46.6	11.6	10.9	13.4	68.0	66.4	74.9
Latvia	70.2	70.0	71.0	15.7	12.4	24.6	69.4	71.1	64.8
Estonia	70.1	71.7	64.3	9.7	8.8	12.8	69.0	69.9	66.8
Denmark	67.9	70.9	50.2	12.7	12.2	15.8	74.2	73.4	81.8
Sweden	65.3	67.7	58.6	11.0	10.4	12.8	77.0	77.7	73.7
Portugal	64.2	66.8	57.5	9.8	8.6	12.6	73.9	73.6	73.9
Cyprus	63.8	68.5	50.7	1.7	1.9	5.3	56.9	51.9	69.3
Hungary	62.7	65.1	55.7	12.9	11.6	17.7	59.1	58.6	61.1
Lithuania	56.5	56.5	56.1	12.6	11.1	16.8	75.2	78.6	65.6
Slovenia	55.7	58.6	46.0	11.1	10.8	11.9	81.4	83.2	77.4
Croatia	54.2	56.1	51.6	23.6	20.1	27.3	79.7	81.3	76.9
Slovakia	54.1	53.1	57.8	18.3	17.7	20.1	65.1	68.8	56.5
Ireland	53.1	56.1	36.0	10.1	9.6	14.6	80.3	80.3	82.0
Spain	52.6	55.3	42.5	5.6	5.5	7.5	66.8	65.8	71.0
Italy	51.1	56.0	42.1	31.2	29.4	34.1	94.7	96.0	91.3
Germany	49.1	49.7	46.4	17.8	16.9	20.6	79.2	82.8	68.0
Iceland	46.8	50.1	28.8	13.5	14.4	9.6	N/A	N/A	N/A
Austria	43.4	45.0	35.8	11.2	9.9	16.4	81.9	83.6	78.1
France	43.1	43.4	40.9	14.9	15.3	14.9	81.0	84.1	71.2
Poland	42.9	42.1	44.6	14.5	12.8	18.5	86.8	87.5	83.1
Czechia	39.7	38.2	42.5	28.5	23.0	40.2	79.3	83.6	69.9
Netherlands	34.4	38.2	14.9	16.2	15.3	23.8	N/A	N/A	N/A
Luxembourg	34.3	36.1	21.1	13.9	12.8	21.4	92.3	94.2	83.5
Malta	27.9	29.1	25.3	10.7	8.4	17.1	79.1	79.3	79.0
Mean	57.1	58.9	49.3	14.6 ^a^	13.0	18.9 ^a^	70.1 ^a^	70.9	68.5 ^a^
SD	15.6	15.6	17.3	6.3 ^a^	5.3	8.2 ^a^	17.4 ^a^	18.2	15.7 ^a^
Median	55.0	56.0	48.5	13.0	12.0	17.0	74.5	76.0	71.0
test statistics ^b^		2.177			−3.194			−1.026	
*p*-value		0.034			0.001			0.305	

Notes: the share calculated out of the total reporting UHCN in a given age group; values sorted by financial reason for total population; SD-standard deviation; N/A-reliable data not available; ^a^ non normal data distribution (checked using the Shapiro–Wilk test); ^b^ *t*-test in the case of financial reasons, Mann–Whitney U test in the case of distance or transportation and waiting list reasons.

**Table 3:** The share of people reporting a given reason of unmet health care needs (UHCN): age groups 65+, 65–74, and 75+ (%).

Country	Financial			Distance or Transportation			Waiting List		
	Age Group								
	65+	65–74	75+	65+	65–74	75+	65+	65–74	75+
Bulgaria	93.7	90.3	97.9	21.3	20.8	21.7	26.7	28.5	24.6
Romania	85.0	86.6	83.8	15.8	13.4	18.6	29.1	27.2	32.0
Latvia	71.0	70.8	71.3	24.6	20.0	30.9	64.8	70.6	57.0
Estonia	64.3	65.9	62.6	12.8	6.8	19.5	66.8	67.1	66.4
Greece	64.3	66.9	61.8	37.0	31.1	44.7	68.5	70.5	66.4
Sweden	58.6	60.5	56.2	12.8	8.3	17.7	73.7	76.7	70.2
Slovakia	57.8	58.9	56.0	20.1	15.9	25.8	56.5	60.3	50.3
Portugal	57.5	58.8	56.0	12.6	11.3	13.8	73.9	73.3	75.0
Lithuania	56.1	58.9	53.3	16.8	7.2	27.4	65.6	68.3	63.3
Hungary	55.7	57.7	52.9	17.7	18.1	16.6	61.1	61.4	60.4
Croatia	51.6	48.7	54.1	27.3	27.8	26.7	76.9	86.1	69.7
Cyprus	50.7	56.4	38.9	5.3	1.3	9.7	69.3	67.9	70.8
Denmark	50.2	46.9	55.2	15.8	15.3	16.2	81.8	86.6	74.3
Norway	50.0	51.7	52.9	25.0	27.6	17.6	37.5	37.9	41.2
Finland	46.6	50.3	40.3	13.4	12.9	13.9	74.9	75.2	74.2
Germany	46.4	49.8	43.0	20.6	14.8	25.8	68.0	69.0	67.2
Slovenia	46.0	49.1	41.9	11.9	7.7	16.7	77.4	81.2	73.0
Poland	44.6	44.2	45.3	18.5	15.1	23.7	83.1	84.1	81.8
Spain	42.5	42.7	43.2	7.5	6.0	8.9	71.0	71.9	70.4
Czechia	42.5	41.3	43.7	40.2	33.8	50.2	69.9	73.2	64.6
Italy	42.1	41.9	42.0	34.1	32.0	36.2	91.3	94.5	88.6
France	40.9	41.6	39.9	14.9	10.7	22.5	71.2	74.1	65.2
Ireland	36.0	39.3	29.6	14.6	15.2	13.8	82.0	82.7	79.9
Austria	35.8	37.0	34.4	16.4	13.0	20.3	78.1	79.3	77.1
Iceland	28.8	28.4	30.1	9.6	4.5	22.2	N/A	N/A	N/A
Malta	25.3	27.6	21.7	17.1	12.4	25.5	79.0	82.8	72.7
Luxembourg	21.1	23.4	17.2	21.4	11.7	38.7	83.5	87.4	77.0
Netherlands	14.9	19.2	8.5	23.8	11.1	36.8	N/A	N/A	N/A
Mean	49.3	50.6	47.6	18.9 ^a^	15.2 ^a^	23.7 ^a^	68.5 ^a^	70.7 ^a^	65.9 ^a^
SD	17.3	16.8	18.8	8.2 ^a^	8.6 ^a^	10.1 ^a^	15.7 ^a^	16.8 ^a^	14.8 ^a^
Median	48.5	49.5	44.5	17.0	13.0	22.0	71.0	73.0	71.0
test statistics ^b^		0.622			−3.337			−1.657	
*p*-value		0.537			<0.001			0.097	

Notes: the share calculated out of the total reporting UHCN in a given age group; values sorted by financial reason for the population 65+; SD-standard deviation; N/A-reliable data not available; ^a^ non normal data distribution (checked using the Shapiro–Wilk test); ^b^ *t*-test in the case of financial reasons, Mann–Whitney U test in the case of distance or transportation and waiting list reasons.

**Table 4:** Comparison of unmet health care needs (UHCN) by educational level.

Age	Measure	Educational Level			F-Value	*p*-Value
		Level 0–2	Level 3–4	Level 5–8		
15–64	mean (SD)	27.6 (9.5)	25.7 (10.6)	23.4 (10.7)	1.184	0.311
	min/max	6.3/45.1	6.5/44.2	4.0/43.7		
65+	mean (SD)	24.5 (9.2)	23.2 (8.7)	21.9 (10.1)	0.533	0.589
	min/max	4.3/44.0	1.8/38.7	1.6/49.3		
65–74	mean (SD)	25.7 (10.1)	23.7 (8.8)	21.9 (10.4)	1.100	0.338
	min/max	5.9/50.3	2.5/42.3	1.7/48.2		
75+	mean (SD)	23.7 (9.4)	22.4 (10.0)	22.2 (10.6)	0.199	0.820
	min/max	3.1/44.1	1.0/41.7	1.3/50.5		

Notes: mean calculated for 28 countries; SD—standard deviation; F-value of the ANOVA test statistic; *p*-value from ANOVA test; educational level 0–2: less than primary, primary and lower secondary education; educational level 3–4: upper secondary and post-secondary non-tertiary education; educational level 5–8: tertiary education.

**Table 5:** Comparison of unmet health care needs by income group.

Age	Measure	Income Group					F-Value	*p*-Value
		First Quintile (1)	Second Quintile (2)	Third Quintile (3)	Fourth Quintile (4)	Fifth Quintile (5)		
15–64	mean (SD)	34.7 (12.3)	29.6(11.6)	25.3 (10.8)	22.8 (10.3)	20.0 (9.6)	7.899	<0.001
	min/max	10.6/62.5	7.5/49.7	4.9/43.4	3.8/39.7	2.4/39.4		
65+	mean (SD)	29.3 (10.8)	24.9 (9.6)	22.7 (9.4)	20.9 (10.0)	18.0 (8.2)	4.625	0.002
	min/max	4.6/54.6	2.4/46.5	2.5/44.3	1.2/41.5	1.0/36.1		
65–74	mean (SD)	32.7 (12.3)	26.6 (10.7)	22.8 (9.9)	21.1 (10.0)	17.1 (7.6)	7.397	<0.001
	min/max	7.6/56.7	4.0/48.0	2.8/49.4	1.6/43.0	0.9/29.5		
75+	mean (SD)	25.5 (11.3)	23.5 (9.5)	23.1 (10.2)	20.8 (10.7)	19.5 (10.6)	1.246	0.295
	min/max	2.7/55.9	1.2/45.4	2.2/45.1	0.0/40.1	1.6/48.6		

Notes: mean calculated for 28 countries; SD—standard deviation; F-value of the ANOVA test statistic; *p*-value from ANOVA test; statistically significant post hoc pair comparisons for income groups (* *p* < 0.05; ** *p* < 0.001): 15–64 ((1)–(3)**, (1)–(4)**, (1)–(5)**, (2)–(4)*, (2)–(5)**); 65 years and over ((1)–(3)*, (1)–(4)*, (1)–(5)**); 65–74 ((1)–(2)*, (1)–(3)**, (1)–(4)**, (1)–(5)**, (2)–(4)*, (2)–(5)*).

**Table 6:** Comparison of unmet health care needs (UHCN) by urbanization group.

Age	Measure	Urbanization Group			F-Value	*p*-Value
		Cities	Towns and Suburbs	Rural Areas		
15–64	mean (SD)	27.9 (11.3)	24.9 (9.7)	23.5 (9.6)	1.360	0.262
	min/max	4.5/46.0	7.8/41.2	4.1/39.8		
65+	mean (SD)	25.2 (9.9)	24.2 (9.5)	22.3 (8.6)	0.688	0.506
	min/max	1.7/43.0	2.1/42.0	3.1/46.7		
65–74	mean (SD)	25.6 (9.9)	24.5 (10.0)	22.8 (9.0)	0.574	0.566
	min/max	2.9/46.9	1.9/41.7	4.0/48.9		
75+	mean (SD)	24.6 (10.6)	23.9 (10.0)	21.7 (9.2)	0.618	0.541
	min/max	0.0/46.8	2.4/42.9	1.9/44.7		

Notes: mean calculated for 28 countries; SD—standard deviation; F-value of the ANOVA test statistic; *p*-value from ANOVA test.

**Table 7:** Comparison of unmet health care needs (UHCN) by level of activity limitation.

Age	Measure	Activity Limitation Level			F-Value	*p*-Value
		None (1)	Moderate (2)	Severe (3)		
15–64	mean (SD)	21.2 (8.7)	39.0 (12.1)	50.6 (12.8)	47.708	<0.001
	min/max	4.5/37.4	11.9/62.7	19.1/70.4		
65+	mean (SD)	17.2 (7.0)	28.0 (10.4)	36.4 (12.0)	25.690	<0.001
	min/max	1.4/32.8	3.6/50.3	9.0/62.4		
65–74	mean (SD)	17.8 (7.2)	30.7 (10.8)	40.7 (13.8)	35.129	<0.001
	min/max	1.8/32.0	4.2/51.4	11.3/67.8		
75+	mean (SD)	16.2 (7.5)	25.1 (10.8)	33.1 (11.9)	19.080	<0.001
	min/max	0.9/37.8	2.7/49.3	6.5/57.7		

Notes: mean calculated for 28 countries; SD—standard deviation; F-value of the ANOVA test statistic; *p*-value from ANOVA test; for the group 65–74, the Welch test was used due to heterogeneity of variance; statistically significant post hoc pair comparisons for activity limitation levels (* *p* < 0.05; ** *p* < 0.001): 15–65 ((1)–(2)**, (1)–(3)**, (2)–(3)**); 65 years and over ((1)–(2)**, (1)–(3)**, (2)–(3)*); 65–74 ((1)–(2)**, (1)–(3)**, (2)–(3)*); 75+ ((1)–(2)*, (1)–(3)**, (2)–(3)*).

## Data Availability

Publicly available datasets were analyzed in this study. These data can be found here: https://ec.europa.eu/eurostat/web/main/data/database (accessed on 30 April 2023).

## Supplementary Materials

The following supporting information can be downloaded at: https://www.mdpi.com/article/10.3390/healthcare11192692/s1, Table S1: Information about UHCN indicators: EHIS indicator and EU-SILC indicator; Questionnaire S1: The European Health Interview Survey questions regarding used indicators; Table S2: Sample size in the European Health Interview Survey—national level (countries analyzed in the study); Table S3: Self-reported UHCN by age and reasons in 2019 (%); Table S4: Self-reported UHCN by age and educational level in 2019 (%); Table S5: Self-reported UHCN by age and income groups in 2019 (%); Table S6: Self-reported UHCN by age and urbanization group in 2019 (%); Table S7: Self-reported UHCN by age and activity limitation level in 2019 (%).
