# Supplementary material for: Unmet Health Care Needs of the Older Population in European Countries Based on Indicators Available in the Eurostat Database
Source: Healthcare (Basel). 2023 Oct 8;11(19):2692. doi: 10.3390/healthcare11192692 (PMC10572618; doi:10.3390/healthcare11192692)
Supplement: Supplementary file 1 [file healthcare-11-02692-s001.zip › healthcare-2603618-supplementary.pdf]

Supplementary Materials

Table S1. Information about UHCN indicators: EHIS indicator and EU-SILC indicator

	**EHIS indicator**	**EU-SILC indicator**
The source of information for indicator construction	questionnaire survey (the European Health Interview Survey)	questionnaire survey (the EU Statistics on Income and Living Conditions)
Name of variable used in the research	UN1A: Unmet need for health care in the past 12 months due to long waiting list;<br>UN1B: Unmet need for health care in the past 12 months due to distance or transportation problems;<br>UN2A: Counld not afford medical examination or treatment in the past 12 months.	PH040: Unmet need for medical examination or treatment;<br>PH050: Main reason for unmet needfor medical examination or treatment.
Type of used information	self-reported, subjective	self-reported, subjective
Construction of indicator:<br>- nominator<br>- denominator	 - number of people with UHCN<br>- number of people in need of health care	 - number of people with UHCN<br>- total population (with and without need of health care)
Scope of health care taken into account	broadly understood, defined according to the System of Health Accounts as individual health care goods and services	separately for a medical and dental care (medical goods excluded)
Population included	15 years and older	16 years and older
"Unmet needs" understanding	delayed or forgone care	forgone care
Number of reasons of UHCN possible to indicate	3	7 + "other reason" option
Reference period	last 12 months	last 12 months

Source: Eurostat. Quality report of the third wave of the European Health Interview Survey. 2022 edition. Publication Office of the European Union. Luxembourg (ref.41 in the main text) and Eurostat. Unmet Health Care Needs Statistics. Available online: https://ec.europa.eu/eurostat/statistics-explained/index.php?title=Unmet_health_care_needs_statistics (accessed 30 April 2023) (ref.14 in the main text)

Questionnaire S1

The European Health Interview Survey questions regarding used indicators:

UN1A: Unmet need for health care in the past 12 months due to long waiting lists

Question: Have you experienced delay in getting health care in the past 12 months because the time needed to obtain an appointment was too long?

1. Yes
2. No
3. No need for health care

UN1B: Unmet need for health care in the past 12 months due to distance or transportation problems

Question: Have you experienced delay in getting health care in the past 12 months due to distance or transportation problems?

1. Yes
2. No
3. No need for health care

UN2: Could not afford [*kind of care: medical care/dental care/prescribed medicines/mental health care*] in the past 12 months

Question: Was there any time in the past 12 months when you needed the following kinds of health care, but could not afford it?

1. Yes
2. No
3. No need for [*kind of care*]

Source: Eurostat. Quality report of the third wave of the European Health Interview Survey. 2022 edition. Publication Office of the European Union. Luxembourg (ref.41 in the main text)

Table S2. Sample size in the European Health Interview Survey – national level (countries analyzed in the study)

Country	Achieved effective sample size	Minimum effective sample size	Ratio of the achieved to minimum effective sample size	Total population (15+) (2019)
Austria (AT)	12 778	6 104	2.09	7 580 083
Bulgaria (BG)	5 273	5 738	0.92	5 995 194
Croatia (HR)	1 986	5 041	0.39	3 488 460
Cyprus (CY)	6 413	3 829	1.67	734 695
Czechia (CZ)	7 612	6 391	1.19	8 956 740
Denmark (DK)	6 629	5 442	1.22	4 848 611
Estonia (EE)	4 833	4 063	1.19	1 107 397
Finland (FI)	6 251	5 384	1.16	4 635 685
France (FR)	N/A	11 705	N/A	55 064 279
Germany (DE)	9 056	12 963	0.70	71 728 398
Greece (GR)	N/A	N/A	N/A	9 187 524
Hungary (HU)	7 004	6 268	1.12	8 351 017
Iceland (IS)	N/A	N/A	N/A	289 174
Ireland (IE)	5 115	5 169	0.99	3 896 482
Italy (IT)	32 597	11 449	2.85	51 944 786
Latvia (LV)	12 066	4 325	2.79	1 614 888
Lithuania (LT)	5 075	4 648	1.09	2 372 327
Luxembourg (LU)	4 416	3 661	1.21	515 287
Malta (MT)	N/A	3 583	N/A	426 055
Netherlands (NL)	7 587	7 376	1.03	14 542 344
Norway (NO)	6 381	5 315	1.20	4 393 254
Poland (PL)	11 088	9 603	1.15	32 139 021
Portugal (PT)	7 236	6 374	1.14	8 869 051
Romania (RO)	N/A	7 656	N/A	16 372 216
Slovakia (SK)	5 527	5 372	1.03	4 592 379
Slovenia (SI)	9 706	4 395	2.21	1 767 202
Spain (ES)	13 882	10 390	1.34	40 006 943
Sweden (SE)	8 131	6 280	1.29	8 410 456

Notes: N/A – data non-available

Source: Eurostat. Quality report of the third wave of the European Health Interview Survey. 2022 edition. Publication Office of the European Union. Luxembourg (ref.41 in the main text)

Table S3. Self-reported UHCN by age and reasons in 2019 (%)

Country	Total UHCN					UHCN due to financial reasons					UHCN due to distance or transportation issues					UHCN due to overlong waiting lists				
	total	15-64	65+	65-74	75+	total	15-64	65+	65-74	75+	total	15-64	65+	65-74	75+	total	15-64	65+	65-74	75+
Austria	24.9	26.2	20.1	20.8	19.2	10.8	11.8	7.2	7.7	6.6	2.8	2.6	3.3	2.7	3.9	20.4	21.9	15.7	16.5	14.8
Bulgaria	15.9	12.9	22.1	20.7	24.0	14.5	11.4	20.7	18.7	23.5	2.9	2.1	4.7	4.3	5.2	4.9	4.4	5.9	5.9	5.9
Croatia	33.0	28.9	40.3	38.8	41.6	17.9	16.2	20.8	18.9	22.5	7.8	5.8	11.0	10.8	11.1	26.3	23.5	31.0	33.4	29.0
Cyprus	5.8	5.4	7.5	7.8	7.2	3.7	3.7	3.8	4.4	2.8	0.1	0.1	0.4	0.1	0.7	3.3	2.8	5.2	5.3	5.1
Czechia	17.9	16.5	21.9	21.3	22.9	7.1	6.3	9.3	8.8	10.0	5.1	3.8	8.8	7.2	11.5	14.2	13.8	15.3	15.6	14.8
Denmark	33.0	36.8	20.9	20.9	21.0	22.4	26.1	10.5	9.8	11.6	4.2	4.5	3.3	3.2	3.4	24.5	27.0	17.1	18.1	15.6
Estonia	37.1	38.5	32.8	33.7	31.8	26.0	27.6	21.1	22.2	19.9	3.6	3.4	4.2	2.3	6.2	25.6	26.9	21.9	22.6	21.1
Finland	36.3	38.4	30.7	29.4	33.0	25.7	29.7	14.3	14.8	13.3	4.2	4.2	4.1	3.8	4.6	24.7	25.5	23.0	22.1	24.5
France	29.5	32.0	21.5	24.3	17.8	12.7	13.9	8.8	10.1	7.1	4.4	4.9	3.2	2.6	4.0	23.9	26.9	15.3	18.0	11.6
Germany	26.9	29.6	19.4	20.3	18.6	13.2	14.7	9.0	10.1	8.0	4.8	5.0	4.0	3.0	4.8	21.3	24.5	13.2	14.0	12.5
Greece	19.7	18.4	23.5	25.1	21.7	14.4	14.1	15.1	16.8	13.4	5.0	3.3	8.7	7.8	9.7	12.5	10.9	16.1	17.7	14.4
Hungary	22.5	23.2	20.3	21.5	18.7	14.1	15.1	11.3	12.4	9.9	2.9	2.7	3.6	3.9	3.1	13.3	13.6	12.4	13.2	11.3
Iceland	32.5	36.1	21.9	26.8	15.3	15.2	18.1	6.3	7.6	4.6	4.4	5.2	2.1	1.2	3.4	33.1	36.0	24.2	29.7	16.1
Ireland	22.8	23.9	17.8	19.1	15.9	12.1	13.4	6.4	7.5	4.7	2.3	2.3	2.6	2.9	2.2	18.3	19.2	14.6	15.8	12.7
Italy	26.6	25.2	29.9	29.1	30.7	13.6	14.1	12.6	12.2	12.9	8.3	7.4	10.2	9.3	11.1	25.2	24.2	27.3	27.5	27.2
Latvia	36.9	37.0	36.6	41.5	31.4	25.9	25.9	26.0	29.4	22.4	5.8	4.6	9.0	8.3	9.7	25.6	26.3	23.7	29.3	17.9
Lithuania	26.2	26.2	26.2	26.5	25.9	14.8	14.8	14.7	15.6	13.8	3.3	2.9	4.4	1.9	7.1	19.7	20.6	17.2	18.1	16.4
Luxembourg	38.8	41.3	26.6	23.9	33.1	13.3	14.9	5.6	5.6	5.7	5.4	5.3	5.7	2.8	12.8	35.8	38.9	22.2	20.9	25.5
Malta	21.5	20.3	25.7	25.0	26.7	6.0	5.9	6.5	6.9	5.8	2.3	1.7	4.4	3.1	6.8	17.0	16.1	20.3	20.7	19.4
Netherlands	15.4	17.0	10.1	9.9	10.6	5.3	6.5	1.5	1.9	0.9	2.5	2.6	2.4	1.1	3.9	17.5	18.9	12.8	13.8	11.5
Norway	11.7	14.3	2.4	2.9	1.7	8.7	10.7	1.2	1.5	0.9	1.3	1.4	0.6	0.8	0.3	3.9	4.8	0.9	1.1	0.7
Poland	29.6	27.3	37.9	37.8	38.0	12.7	11.5	16.9	16.7	17.2	4.3	3.5	7.0	5.7	9.0	25.7	23.9	31.5	31.8	31.1
Portugal	39.9	39.4	41.4	40.8	42.0	25.6	26.3	23.8	24.0	23.5	3.9	3.4	5.2	4.6	5.8	29.5	29.0	30.6	29.9	31.5
Romania	13.8	10.4	24.7	24.6	24.7	12.1	9.2	21.0	21.3	20.7	1.7	1.0	3.9	3.3	4.6	3.6	2.5	7.2	6.7	7.9
Slovakia	10.9	9.6	15.4	15.1	15.9	5.9	5.1	8.9	8.9	8.9	2.0	1.7	3.1	2.4	4.1	7.1	6.6	8.7	9.1	8.0
Slovenia	28.0	29.7	22.6	23.4	21.5	15.6	17.4	10.4	11.5	9.0	3.1	3.2	2.7	1.8	3.6	22.8	24.7	17.5	19.0	15.7
Spain	19.6	19.9	18.6	19.9	16.9	10.3	11.0	7.9	8.5	7.3	1.1	1.1	1.4	1.2	1.5	13.1	13.1	13.2	14.3	11.9
Sweden	31.7	33.7	26.6	26.6	26.5	20.7	22.8	15.6	16.1	14.9	3.5	3.5	3.4	2.2	4.7	24.4	26.2	19.6	20.4	18.6

Notes: sorted alphabetically by countries

Table S4. Self-reported UHCN by age and educational level in 2019 (%)

Country	Age group																							
	65+						65-74						75+						15-64					
	Educational levels						Educational levels						Educational levels						Educational levels					
	Levels 0-2	Levels 3-4	Levels 5-8	min	max	range[1]	Levels 0-2	Levels 3-4	Levels 5-8	min	max	range[1]	Levels 0-2	Levels 3-4	Levels 5-8	min	max	range[1]	Levels 0-2	Levels 3-4	Levels 5-8	min	max	range[1]
Austria	20.2	19.6	20.8	19.6	20.8	1.2	21.6	19.8	22.4	19.8	22.4	2.6	19.3	19.4	18.5	18.5	19.4	0.9	24.6	26.5	26.5	24.6	26.5	1.9
Bulgaria	26.3	21.7	16.1	16.1	26.3	10.2	28.7	19.8	14.8	14.8	28.7	13.9	24.6	25.3	18.8	18.8	25.3	6.5	22.3	11.5	9.4	9.4	22.3	12.9
Croatia	38.5	38.7	49.3	38.5	49.3	10.8	37.7	36.5	48.2	36.5	48.2	11.7	39.0	41.7	50.5	39.0	50.5	11.5	29.2	29.7	27.0	27.0	29.7	2.7
Cyprus	8.5	7.4	3.8	3.8	8.5	4.7	10.6	5.6	3.6	3.6	10.6	7.0	6.3	10.9	4.3	4.3	10.9	6.6	6.3	6.5	4.0	4.0	6.5	2.5
Czechia	20.6	22.2	22.1	20.6	22.2	1.6	16.3	22.2	20.9	16.3	22.2	5.9	24.4	22.2	24.1	22.2	24.4	2.2	20.0	16.5	14.8	14.8	20.0	5.2
Denmark	22.1	20.7	20.0	20.0	22.1	2.1	23.2	19.5	20.6	19.5	23.2	3.7	21.2	22.5	18.7	18.7	22.5	3.8	43.5	39.9	30.8	30.8	43.5	12.7
Estonia	32.5	30.1	36.4	30.1	36.4	6.3	34.5	31.9	35.8	31.9	35.8	3.9	31.5	28.0	37.3	28.0	37.3	9.3	35.3	40.0	37.9	35.3	40.0	4.7
Finland	32.8	31.4	27.0	27.0	32.8	5.8	32.1	31.7	23.6	23.6	32.1	8.5	33.5	30.6	34.3	30.6	34.3	3.7	42.9	44.2	29.0	29.0	44.2	15.2
France	21.0	23.7	22.2	21.0	23.7	2.7	24.0	25.4	24.4	24.0	25.4	1.4	17.6	20.4	17.7	17.6	20.4	2.8	28.2	31.4	37.0	28.2	37.0	8.8
Germany	21.3	19.1	17.5	17.5	21.3	3.8	24.8	20.1	17.0	17.0	24.8	7.8	19.5	18.1	18.0	18.0	19.5	1.5	33.1	30.0	26.1	26.1	33.1	7.0
Greece	27.0	19.6	15.0	15.0	27.0	12.0	30.4	23.4	14.4	14.4	30.4	16.0	24.6	12.1	16.2	12.1	24.6	12.5	28.2	19.5	10.7	10.7	28.2	17.5
Hungary	15.5	20.8	25.8	15.5	25.8	10.3	15.4	23.5	21.8	15.4	23.5	8.1	15.6	15.0	32.7	15.0	32.7	17.7	23.2	22.7	24.1	22.7	24.1	1.4
Iceland	20.4	23.8	22.0	20.4	23.8	3.4	25.2	29.7	24.5	24.5	29.7	5.2	16.0	13.6	16.4	13.6	16.4	2.8	37.4	36.8	34.4	34.4	37.4	3.0
Ireland	18.9	18.0	15.3	15.3	18.9	3.6	19.9	21.0	15.6	15.6	21.0	5.4	17.6	12.5	14.5	12.5	17.6	5.1	23.4	26.3	21.6	21.6	26.3	4.7
Italy	30.2	29.8	26.5	26.5	30.2	3.7	29.5	28.9	26.6	26.6	29.5	2.9	30.8	31.7	26.4	26.4	31.7	5.3	28.2	24.2	21.8	21.8	28.2	6.4
Latvia	39.0	37.8	31.2	31.2	39.0	7.8	50.3	42.3	34.7	34.7	50.3	15.6	34.3	31.1	26.3	26.3	34.3	8.0	38.2	39.8	31.8	31.8	39.8	8.0
Lithuania	27.4	25.9	25.8	25.8	27.4	1.6	30.0	26.6	25.3	25.3	30.0	4.7	26.8	25.0	26.7	25.0	26.8	1.8	22.4	24.3	29.3	22.4	29.3	6.9
Luxembourg	29.5	26.6	23.0	23.0	29.5	6.5	29.7	24.2	20.0	20.0	29.7	9.7	29.0	33.2	31.6	29.0	33.2	4.2	34.9	40.1	43.7	34.9	43.7	8.8
Malta	25.4	31.5	22.9	22.9	31.5	8.6	24.7	29.8	22.8	22.8	29.8	7.0	26.5	35.0	22.9	22.9	35.0	12.1	22.2	17.8	19.2	17.8	22.2	4.4
Netherlands	9.6	10.9	10.3	9.6	10.9	1.3	8.3	13.0	8.9	8.3	13.0	4.7	11.1	7.3	12.9	7.3	12.9	5.6	17.8	16.9	16.4	16.4	17.8	1.4
Norway	4.3	1.8	1.6	1.6	4.3	2.7	5.9	2.5	1.7	1.7	5.9	4.2	3.1	1.0	1.3	1.0	3.1	2.1	19.0	14.5	11.1	11.1	19.0	7.9
Poland	36.0	37.7	41.8	36.0	41.8	5.8	36.6	36.9	43.2	36.6	43.2	6.6	35.6	39.4	39.6	35.6	39.6	4.0	22.0	27.4	29.2	22.0	29.2	7.2
Portugal	44.0	30.6	21.9	21.9	44.0	22.1	43.9	32.3	23.0	23.0	43.9	20.9	44.1	25.1	20.2	20.2	44.1	23.9	45.1	35.8	32.0	32.0	45.1	13.1
Romania	26.7	22.8	17.9	17.9	26.7	8.8	27.8	22.8	18.7	18.7	27.8	9.1	25.9	22.7	16.5	16.5	25.9	9.4	15.8	10.0	5.1	5.1	15.8	10.7
Slovakia	19.5	14.1	14.1	14.1	19.5	5.4	17.6	15.1	11.9	11.9	17.6	5.7	20.7	12.0	19.7	12.0	20.7	8.7	17.0	9.9	6.0	6.0	17.0	11.0
Slovenia	18.9	25.3	24.8	18.9	25.3	6.4	18.5	20.6	27.7	18.5	27.7	9.2	19.1	32.3	19.4	19.1	32.3	13.2	28.5	23.0	31.4	23.0	31.4	8.4
Spain	20.5	15.5	11.0	11.0	20.5	9.5	22.7	16.4	12.1	12.1	22.7	10.6	18.3	13.5	8.8	8.8	18.3	9.5	23.9	20.6	14.5	14.5	23.9	9.4
Sweden	29.3	23.8	27.3	23.8	29.3	5.5	30.9	23.0	28.0	23.0	30.9	7.9	28.0	24.8	26.2	24.8	28.0	3.2	40.6	33.5	30.2	30.2	40.6	10.4
**min**	**4.3**	**1.8**	**1.6**				**5.9**	**2.5**	**1.7**				**3.1**	**1.0**	**1.3**				**6.3**	**6.5**	**4.0**			
**max**	**44.0**	**38.7**	**49.3**				**50.3**	**42.3**	**48.2**				**44.1**	**41.7**	**50.5**				**45.1**	**44.2**	**43.7**			
**range (max-min)**	**39.7**	**36.9**	**47.7**				**44.4**	**39.8**	**46.5**				**41.0**	**40.7**	**49.2**				**38.8**	**37.7**	**37.9**			

Notes: sorted alphabetically by countries; Educational levels 0-2: Less than primary, primary and lower secondary education; Educational levels 3-4: Upper secondary and post-secondary non-tertiary education; Educational levels 5-8: Tertiary education; [1] difference between maximum and minimum values

Table S5. Self-reported UHCN by age and income groups in 2019 (%)

Country	Age group																							
	65+						65-74						75+						15-64					
	Income quintile group					range[1]	Income quintile group					range[1]	Income quintile group					range[1]	Income quintile group					range[1]
	1.	2.	3.	4.	5.		1.	2.	3.	4.	5.		1.	2.	3.	4.	5.		1.	2.	3.	4.	5.	
Austria	22.0	21.8	19.0	13.9	15.5	8.1	24.0	22.6	21.0	13.3	15.2	10.7	20.6	20.9	15.9	15.0	15.9	5.9	33.3	29.5	24.7	22.8	22.4	10.9
Bulgaria	29.8	22.5	20.1	13.7	19.8	16.1	35.3	20.6	16.8	12.8	18.5	22.5	25.4	24.5	26.0	15.5	23.9	10.5	24.5	19.3	13.9	8.6	10	15.9
Croatia	39.9	37.6	43.3	41.5	36.1	7.2	40.1	45.6	49.4	43.0	25.9	23.5	39.7	31.8	38.1	40.1	48.6	16.8	41.4	31.5	30.3	32.4	31.1	11.1
Cyprus	10.7	6.5	8.5	4.5	4.5	6.2	11.7	7.2	8.2	5.4	4.1	7.6	9.7	5.7	8.9	2.6	5.7	7.1	10.6	7.5	4.9	3.8	2.4	8.2
Czechia	24.9	22.1	20.1	17.6	18.6	7.3	25.5	19.3	21.6	18.2	19.6	7.3	24.4	26.2	16.8	15.5	13.5	12.7	24.4	16.4	14.8	15	14.3	10.1
Denmark	23.1	19.5	15.1	18.0	19.4	8.0	25.8	20.1	13.4	17.6	21.0	12.4	20.8	18.6	18.8	19.6	16.1	4.7	54.9	43.5	33.7	28.6	22.9	32.0
Estonia	33.2	34.0	32.3	37.7	15.2	22.5	35.2	37.7	28.6	39.4	15.6	23.8	31.8	30.6	39.1	33.2	N/A	8.5	49.7	42.2	43.4	33.5	32	17.7
Finland	32.6	35.1	30.0	27.0	24.8	10.3	36.3	34.1	29.7	25.0	24.8	11.5	26.3	36.5	30.6	31.8	24.8	11.7	46.7	43.1	37.5	34.1	24.8	21.9
France	28.4	22.4	18.3	19.5	20.6	10.1	37.4	25.4	19.4	22.7	21.0	18.0	18.7	19.0	16.6	14.7	20.0	5.3	35.3	32.3	31.1	30.2	30.6	5.1
Germany	23.4	22.2	19.7	16.8	13.5	9.9	25.2	23.0	20.0	17.4	15.2	10.0	21.6	21.4	19.6	16.3	11.6	10.0	37.1	32.5	27.9	27.3	23.5	13.6
Greece	37.8	31.3	23.9	16.0	10.4	27.4	47.0	36.6	24.1	19.5	2.5	44.5	27.6	27.2	23.6	10.7	23.6	16.9	39.8	24	16.9	9.7	3.4	36.4
Hungary	22.1	17.0	19.6	24.6	19.0	7.6	24.6	17.4	21.7	25.6	16.4	9.2	18.2	16.5	16.7	23.0	26.5	10.0	27.6	21.9	21.1	20.8	24.6	6.8
Iceland	38.7	17.2	21.1	26.0	24.7	21.5	53.1	24.5	22.0	24.7	26.8	31.1	7.5	10.9	20.4	N/A	N/A	12.9	33.7	49.7	43.1	34.9	24.1	25.6
Ireland	22.8	19.8	12.2	3.3	5.2	19.5	27.0	23.1	13.2	4.2	N/A	22.8	17.6	14.7	9.7	N/A	N/A	7.9	35.5	29.1	22	23.8	17	18.5
Italy	37.8	33.2	28.7	27.0	26.1	11.7	36.6	35.0	28.0	26.2	24.1	12.5	39.1	31.7	29.4	28.0	28.8	11.1	33.4	27.4	25.1	21.7	19.4	14.0
Latvia	44.6	36.6	28.3	21.8	17.3	27.3	56.7	44.6	27.7	24.0	15.4	41.3	35.8	28.2	29.4	16.6	20.6	19.2	62.5	43.5	34.3	30.8	26	36.5
Lithuania	29.1	26.4	24.6	24.6	20.5	8.6	32.1	29.3	21.8	23.9	N/A	10.3	26.9	23.1	28.4	25.9	N/A	5.3	37	31.7	25.6	25.2	26.1	11.8
Luxembourg	31.7	26.8	23.3	16.3	36.2	19.9	33.1	25.6	18.0	13.3	33.7	20.4	28.4	28.7	39.1	29.3	N/A	10.7	49.3	44.3	37.2	39.7	39.4	12.1
Malta	25.2	25.6	27.9	27.0	19.0	8.9	24.7	23.4	31.2	24.7	21.6	9.6	26.2	28.7	21.8	31.1	N/A	9.3	24.2	27	18.9	16.8	19.6	10.2
Netherlands	13.1	13.8	11.3	8.1	9.1	5.7	13.7	13.3	11.5	7.1	9.4	6.6	N/A	14.6	11.1	9.6	8.8	5.8	23.4	22.5	13.7	13	11.8	11.6
Norway	4.6	2.4	2.5	1.2	1.0	3.6	7.6	4.0	2.8	1.6	0.9	6.7	2.7	1.2	2.2	0.0	1.6	2.7	26.5	17	12.9	8.3	7.4	19.1
Poland	45.8	38.2	37.1	34.1	33.5	12.3	46.7	37.8	37.9	34.5	29.5	17.2	45.0	38.7	35.9	33.4	41.1	11.6	36	29.4	29.2	26.2	24.4	11.6
Portugal	54.6	46.5	44.3	37.6	22.5	32.1	53.4	48.0	43.6	36.5	24.8	28.6	55.9	45.4	45.1	38.9	18.3	37.6	46.5	44.7	41.5	38.3	26.5	20.0
Romania	30.2	25.2	24.3	23.2	14.5	15.7	33.0	25.5	24.8	21.9	14.1	18.9	28.1	24.8	23.5	25.6	15.1	13.0	17.9	11.3	11.4	9	5.5	12.4
Slovakia	12.9	20.9	15.2	10.0	14.0	10.9	11.7	20.2	14.3	11.9	14.5	8.5	15.1	21.8	16.5	7.0	12.1	14.8	15	11.5	8	8.6	6.4	8.6
Slovenia	24.6	21.6	21.2	24.1	21.8	3.4	26.0	23.1	21.3	25.4	23.6	4.7	23.7	19.5	20.8	21.9	19.6	4.2	30.8	31	30.2	29.4	28.3	2.7
Spain	26.3	21.0	19.3	15.0	14.8	11.5	31.9	23.6	21.6	16.7	14.9	17.0	20.6	19.3	16.9	12.5	14.5	8.1	26.4	22.2	21.1	16.2	12.4	14.0
Sweden	34.4	29.9	25.3	22.2	19.7	14.7	40.7	34.3	24.2	22.0	20.1	20.6	30.5	27.1	26.7	22.8	18.7	11.8	48.8	44.1	34.2	29.6	23.3	25.5
**min**	**4.6**	**2.4**	**2.5**	**1.2**	**1.0**		**7.6**	**4.0**	**2.8**	**1.6**	**0.9**		**2.7**	**1.2**	**2.2**	**0.0**	**1.6**		**10.6**	**7.5**	**4.9**	**3.8**	**2.4**	
**max**	**54.6**	**46.5**	**44.3**	**41.5**	**36.2**		**56.7**	**48.0**	**49.4**	**43.0**	**33.7**		**55.9**	**45.4**	**45.1**	**40.1**	**48.6**		**62.5**	**49.7**	**43.4**	**39.7**	**39.4**	
**range (max-min)**	**50.0**	**44.1**	**41.8**	**40.3**	**35.2**		**49.1**	**44.0**	**46.6**	**41.4**	**32.8**		**53.2**	**44.2**	**42.9**	**40.1**	**47.0**		**51.9**	**42.2**	**38.5**	**35.9**	**37.0**	

Notes: sorted alphabetically by countries; [1] difference between maximum and minimum values; N/A: data not available

Table S6. Self-reported UHCN by age and urbanization group in 2019 (%)

Country	Age group: 65+						65-74						75+						15-64					
	Urbanization group						Urbanization group						Urbanization group						Urbanization group					
	**Cities**	**Towns/ suburbs**	**Rural**	min	max	range[1]	**Cities**	**Towns/ suburbs**	**Rural**	min	max	range[1]	**Cities**	**Towns/ suburbs**	**Rural**	min	max	range[1]	**Cities**	**Towns/ suburbs**	**Rural**	min	max	range[1]
Austria	19.8	19.3	20.7	19.3	20.7	1.4	19.9	18.8	22.9	18.8	22.9	4.1	19.7	19.9	18.3	18.3	19.9	1.6	31.5	24.8	23.1	23.1	31.5	8.4
Bulgaria	23.1	18.2	25.8	18.2	25.8	7.6	20.6	16.4	28.4	16.4	28.4	12	27.4	20.4	23.0	20.4	27.4	7	12.6	10.6	16.8	10.6	16.8	6.2
Croatia	43.0	42.0	35.3	35.3	43	7.7	38.6	41.1	36.8	36.8	41.1	4.3	46.8	42.9	34.0	34	46.8	12.8	30.6	31.6	24.5	24.5	31.6	7.1
Cyprus	6.8	13.3	5.2	5.2	13.3	8.1	7.1	12.5	5.7	5.7	12.5	6.8	6.4	14.8	4.5	4.5	14.8	10.3	4.5	9.9	4.1	4.1	9.9	5.8
Czechia	21.0	22.9	21.9	21	22.9	1.9	21.3	22.5	20.2	20.2	22.5	2.3	20.7	23.5	24.9	20.7	24.9	4.2	19.1	17.2	13.8	13.8	19.1	5.3
Denmark	21.7	20.9	20.5	20.5	21.7	1.2	22.3	21.0	19.9	19.9	22.3	2.4	20.8	20.7	21.4	20.7	21.4	0.7	38.3	36.7	35.4	35.4	38.3	2.9
Estonia	35.4	39.8	27.0	27	39.8	12.8	36.0	41.7	28.2	28.2	41.7	13.5	34.7	37.7	25.7	25.7	37.7	12	41.2	34.7	34.1	34.1	41.2	7.1
Finland	33.8	32.7	25.4	25.4	33.8	8.4	34.6	30.8	22.9	22.9	34.6	11.7	32.6	36.5	29.8	29.8	36.5	6.7	43.	37.2	31.1	31.1	43.0	11.9
France	22.2	21.2	21.2	21.2	22.2	1	26.1	23.3	23.6	23.3	26.1	2.8	17.5	18.2	17.8	17.5	18.2	0.7	34.8	29.6	30.7	29.6	34.8	5.2
Germany	19.5	19.3	19.3	19.3	19.5	0.2	20.3	19.9	21.8	19.9	21.8	1.9	18.8	18.8	16.8	16.8	18.8	2	32.8	27.2	26.7	26.7	32.8	6.1
Greece	23.9	22.8	23.1	22.8	23.9	1.1	25.5	25.6	24.1	24.1	25.6	1.5	22.0	19.2	22.3	19.2	22.3	3.1	19.	16.5	18.6	16.5	19.0	2.5
Hungary	23.6	19.3	17.4	17.4	23.6	6.2	23.4	21.9	18.8	18.8	23.4	4.6	23.7	15.6	15.2	15.2	23.7	8.5	27.4	23.4	18.4	18.4	27.4	9.0
Iceland	21.6	27.6	18.3	18.3	27.6	9.3	26.7	37.6	18.0	18	37.6	19.6	13.8	15.5	18.5	13.8	18.5	4.7	35.6	38.	35.9	35.6	38.0	2.4
Ireland	19.4	16.1	17.3	16.1	19.4	3.3	21.2	15.1	18.9	15.1	21.2	6.1	16.4	17.4	15.0	15	17.4	2.4	26.6	24.1	21.4	21.4	26.6	5.2
Italy	29.9	30.7	28.3	28.3	30.7	2.4	28.1	29.7	28.9	28.1	29.7	1.6	31.9	31.7	27.8	27.8	31.9	4.1	25.9	24.7	25.5	24.7	25.9	1.2
Latvia	40.5	35.1	32.3	32.3	40.5	8.2	46.9	38.1	36.0	36	46.9	10.9	33.3	32.3	28.3	28.3	33.3	5	41.4	31.5	34.7	31.5	41.4	9.9
Lithuania	31.7	24.5	20.3	20.3	31.7	11.4	32.7	20.7	21.3	20.7	32.7	12	30.6	28.7	19.3	19.3	30.6	11.3	29.5	22.3	24.2	22.3	29.5	7.2
Luxembourg	27.3	27.5	25.1	25.1	27.5	2.4	25.1	25.1	22.0	22	25.1	3.1	30.5	33.5	34.4	30.5	34.4	3.9	46.	41.2	38.7	38.7	46.0	7.3
Malta	28.0	23.1	22.5	22.5	28	5.5	27.8	22.4	18.6	18.6	27.8	9.2	28.3	24.3	n/a	24.3	28.3	4	21.5	19.7	12.4	12.4	21.5	9.1
Netherlands	9.5	10.5	11.7	9.5	11.7	2.2	8.6	10.9	12.0	8.6	12	3.4	10.8	9.9	11.3	9.9	11.3	1.4	19.5	13.7	13.7	13.7	19.5	5.8
Norway	1.7	2.1	3.1	1.7	3.1	1.4	2.9	1.9	4.0	1.9	4	2.1	0	2.4	1.9	0	2.4	2.4	14.8	14.2	13.8	13.8	14.8	1.0
Poland	42.9	36.9	33.4	33.4	42.9	9.5	42.6	36.8	33.3	33.3	42.6	9.3	43.3	37.1	33.5	33.5	43.3	9.8	33.1	26.3	23.	23.0	33.1	10.1
Portugal	38.2	41.0	46.7	38.2	46.7	8.5	36.9	39.7	48.9	36.9	48.9	12	39.8	42.6	44.7	39.8	44.7	4.9	40.	38.4	39.8	38.4	40.0	1.6
Romania	25.8	26.2	22.8	22.8	26.2	3.4	24.2	27.6	22.9	22.9	27.6	4.7	28.3	24.2	22.7	22.7	28.3	5.6	7.5	12.2	11.7	7.5	12.2	4.7
Slovakia	22.9	11.2	14.7	11.2	22.9	11.7	23.6	10.3	14.4	10.3	23.6	13.3	21.9	12.7	15.1	12.7	21.9	9.2	12.8	7.8	9.4	7.8	12.8	5.0
Slovenia	26.4	24.5	19.8	19.8	26.4	6.6	26.3	25.9	20.5	20.5	26.3	5.8	26.6	22.8	19.1	19.1	26.6	7.5	37.1	30.7	26.6	26.6	37.1	10.5
Spain	17.1	21.0	19.3	17.1	21	3.9	18.0	23.1	20.9	18	23.1	5.1	15.9	18.3	17.7	15.9	18.3	2.4	19.7	20.8	18.4	18.4	20.8	2.4
Sweden	27.7	26.6	25.0	25	27.7	2.7	29.3	25.2	25.8	25.2	29.3	4.1	26.1	28.3	23.9	23.9	28.3	4.4	35.1	33.1	30.9	30.9	35.1	4.2
**min**	**1.7**	**2.1**	**3.1**				**2.9**	**1.9**	**4**				**0**	**2.4**	**1.9**				**4.5**	**7.8**	**4.1**			
**max**	**43**	**42**	**46.7**				**46.9**	**41.7**	**48.9**				**46.8**	**42.9**	**44.7**				**46.0**	**41.2**	**39.8**			
**range (max-min)**	**41.3**	**39.9**	**43.6**				**44**	**39.8**	**44.9**				**46.8**	**40.5**	**42.8**				**41.5**	**33.4**	**35.7**			

Notes: sorted alphabetically by countries; [1] difference between maximum and minimum values

Table S7. Self-reported UHCN by age and activity limitation level in 2019 (%)

Country	Age group																							
	65+						65-74						75+						15-64					
	Activity limitation						Activity limitation						Activity limitation						Activity limitation					
	None	Mode-rate	Severe	min	max	range[1]	None	Mode-rate	Severe	min	max	range[1]	None	Mode-rate	Severe	min	max	range[1]	None	Mode-rate	Severe	min	max	range[1]
Austria	15.3	22.0	28.7	15.3	28.7	13.4	15.9	24.6	35.9	15.9	35.9	20.0	14.3	19.3	25.7	14.3	25.7	11.4	22.4	35.4	47.3	22.4	47.3	24.9
Bulgaria	13.2	28.5	33.9	13.2	33.9	20.7	12.9	29.4	38.7	12.9	38.7	25.8	14.0	27.5	31.4	14	31.4	17.4	10.4	26.7	26.0	10.4	26.7	16.3
Croatia	32.8	37.7	50.7	32.8	50.7	17.9	30.3	39.5	51.8	30.3	51.8	21.5	37.8	36.0	50.2	36	50.2	14.2	23.6	39.0	51.1	23.6	51.1	27.5
Cyprus	5.1	9.6	12.7	5.1	12.7	7.6	5.4	12.9	13.4	5.4	13.4	8.0	4.7	6.1	12.3	4.7	12.3	7.6	4.5	11.9	19.1	4.5	19.1	14.6
Czechia	15.7	23.1	35.3	15.7	35.3	19.6	16.0	25.4	34.2	16	34.2	18.2	14.6	20.4	36.0	14.6	36	21.4	13.0	25.7	45.3	13.0	45.3	32.3
Denmark	14.2	26.4	39.5	14.2	39.5	25.3	15.0	28.1	44.6	15	44.6	29.6	12.5	24.7	35.3	12.5	35.3	22.8	31.4	48.5	68.1	31.4	68.1	36.7
Estonia	23.7	36.6	38.7	23.7	38.7	15.0	26.5	39.1	38.3	26.5	39.1	12.6	18.7	34.1	38.9	18.7	38.9	20.2	31.8	48.8	61.6	31.8	61.6	29.8
Finland	21.2	37.8	62.4	21.2	62.4	41.2	20.9	38.6	67.8	20.9	67.8	46.9	21.9	36.7	57.7	21.9	57.7	35.8	30.7	52.8	66.4	30.7	66.4	35.7
France	18.1	24.7	28.6	18.1	28.6	10.5	20.6	29.1	34.1	20.6	34.1	13.5	13.7	19.7	24.6	13.7	24.6	10.9	28.9	42.8	48.1	28.9	48.1	19.2
Germany	12.3	22.4	34.2	12.3	34.2	21.9	13.4	24.0	41.7	13.4	41.7	28.3	11.0	21.4	29.6	11	29.6	18.6	23.2	40.8	53.0	23.2	53.0	29.8
Greece	17.6	36.7	36.4	17.6	36.7	19.1	19.2	42.4	50.6	19.2	50.6	31.4	15.6	32.3	30.1	15.6	32.3	16.7	17.0	32.9	39.7	17.0	39.7	22.7
Hungary	16.9	21.6	27.3	16.9	27.3	10.4	16.7	26.5	28.1	16.7	28.1	11.4	17.3	15.3	26.8	15.3	26.8	11.5	19.2	38.3	43.6	19.2	43.6	24.4
Iceland	15.1	24.9	43.0	15.1	43	27.9	17.1	32.4	57.4	17.1	57.4	40.3	12.3	15.1	24.5	12.3	24.5	12.2	29.0	51.8	70.4	29.0	70.4	41.4
Ireland	13.5	21.7	35.7	13.5	35.7	22.2	13.5	28.1	44.7	13.5	44.7	31.2	13.4	13.8	28.6	13.4	28.6	15.2	20.6	43.6	45.1	20.6	45.1	24.5
Italy	24.0	33.4	40.1	24	40.1	16.1	23.2	36.1	41.6	23.2	41.6	18.4	25.2	31.2	39.5	25.2	39.5	14.3	22.1	39.7	47.3	22.1	47.3	25.2
Latvia	22.1	38.4	48.4	22.1	48.4	26.3	24.6	47.7	57.8	24.6	57.8	33.2	16.5	28.8	43.5	16.5	43.5	27.0	27.7	48.7	65.0	27.7	65.0	37.3
Lithuania	21.1	32.3	31.7	21.1	32.3	11.2	23.0	34.1	30.2	23	34.1	11.1	18.1	31.0	32.3	18.1	32.3	14.2	23.0	43.0	50.8	23.0	50.8	27.8
Luxembourg	19.2	35.8	52.4	19.2	52.4	33.2	17.2	34.6	n/a	17.2	34.6	17.4	26.1	38.3	47.5	26.1	47.5	21.4	37.4	57.3	68.7	37.4	68.7	31.3
Malta	19.4	32.3	41.7	19.4	41.7	22.3	19.8	33.8	39.1	19.8	39.1	19.3	18.4	30.6	44.0	18.4	44	25.6	17.9	32.1	45.1	17.9	45.1	27.2
Netherlands	6.6	11.4	21.7	6.6	21.7	15.1	6.7	11.6	23.5	6.7	23.5	16.8	6.5	11.2	20.3	6.5	20.3	13.8	12.3	24.7	48.0	12.3	48.0	35.7
Norway	1.4	3.6	9.0	1.4	9.0	7.6	1.8	4.2	11.3	1.8	11.3	9.5	0.9	2.7	6.5	0.9	6.5	5.6	11.3	23.7	38.1	11.3	38.1	26.8
Poland	28.3	43.8	47.6	28.3	47.6	19.3	29.1	45.7	53.0	29.1	53	23.9	26.0	41.5	44.4	26	44.4	18.4	22.5	45.6	57.9	22.5	57.9	35.4
Portugal	29.6	50.3	56.2	29.6	56.2	26.6	32.0	51.4	62.7	32	62.7	30.7	25.4	49.3	53.9	25.4	53.9	28.5	34.1	62.7	64.5	34.1	64.5	30.4
Romania	16.3	27.6	40.3	16.3	40.3	24.0	16.7	29.6	53.0	16.7	53	36.3	15.5	25.1	35.3	15.5	35.3	19.8	7.8	31.4	47.5	7.8	47.5	39.7
Slovakia	8.9	15.9	23.2	8.9	23.2	14.3	9.1	15.4	29.0	9.1	29	19.9	8.0	16.7	19.4	8	19.4	11.4	6.2	15.8	32.6	6.2	32.6	26.4
Slovenia	16.2	25.9	30.1	16.2	30.1	13.9	17.3	29.2	29.4	17.3	29.4	12.1	14.2	21.8	30.5	14.2	30.5	16.3	25.4	39.3	52.6	25.4	52.6	27.2
Spain	13.7	24.2	31.5	13.7	31.5	17.8	14.8	29.0	40.5	14.8	40.5	25.7	11.8	20.6	25.9	11.8	25.9	14.1	16.2	37.5	51.4	16.2	51.4	35.2
Sweden	18.8	34.0	37.2	18.8	37.2	18.4	18.5	37.1	45.9	18.5	45.9	27.4	19.3	31.0	32.1	19.3	32.1	12.8	25.0	50.6	63.8	25.0	63.8	38.8
**min**	**1.4**	**3.6**	**9**				**1.8**	**4.2**	**11.3**				**0.9**	**2.7**	**6.5**				**4.5**	**11.9**	**19.1**			
**max**	**32.8**	**50.3**	**62.4**				**32**	**51.4**	**67.8**				**37.8**	**49.3**	**57.7**				**37.4**	**62.7**	**70.4**			
**range (max-min)**	**31.4**	**46.7**	**53.4**				**30.2**	**47.2**	**56.5**				**36.9**	**46.6**	**51.2**				**32.9**	**50.8**	**51.3**			

Notes: sorted alphabetically by countries; [1] difference between maximum and minimum values
